# Supplementary material for: Global Transcriptomic Analysis of Zebrafish Glucagon Receptor Mutant Reveals Its Regulated Metabolic Network
Source: Int J Mol Sci. 2020 Jan 22;21(3):724. doi: 10.3390/ijms21030724 (PMC7037442; doi:10.3390/ijms21030724)
Supplement: Supplementary file 1 [file ijms-21-00724-s001.zip › Supplementary files 20191222/Supplemental Figure 1 Legend.docx]

**Supplemental Figure 1 Legned**

**Supplemental Figure 1, The morphology and body length of wild type and gcgr mutant fish.**

**A,** The representative images of *gcgr^-/-^* and wild type zebrafish in different stages. Wild type and *gcgr^-/-^* zebrafish embryos were monitored from 1 dpf to 7dpf, and images were taken under 1,3,5,7 dpf. B, the body length comparison between wild type and *gcgr^-/-^* zebrafish from 3 dpf to 7 dpf. The larval body length was measured using plotting scale tool in the Leica Application Suite X (LAS X) software. Results are means with standard errors (n=15). NS, no significance.
