# Supplementary figures and images for: Global Transcriptomic Analysis of Zebrafish Glucagon Receptor Mutant Reveals Its Regulated Metabolic Network
Source: Int J Mol Sci. 2020 Jan 22;21(3):724. doi: 10.3390/ijms21030724 (PMC7037442; doi:10.3390/ijms21030724)

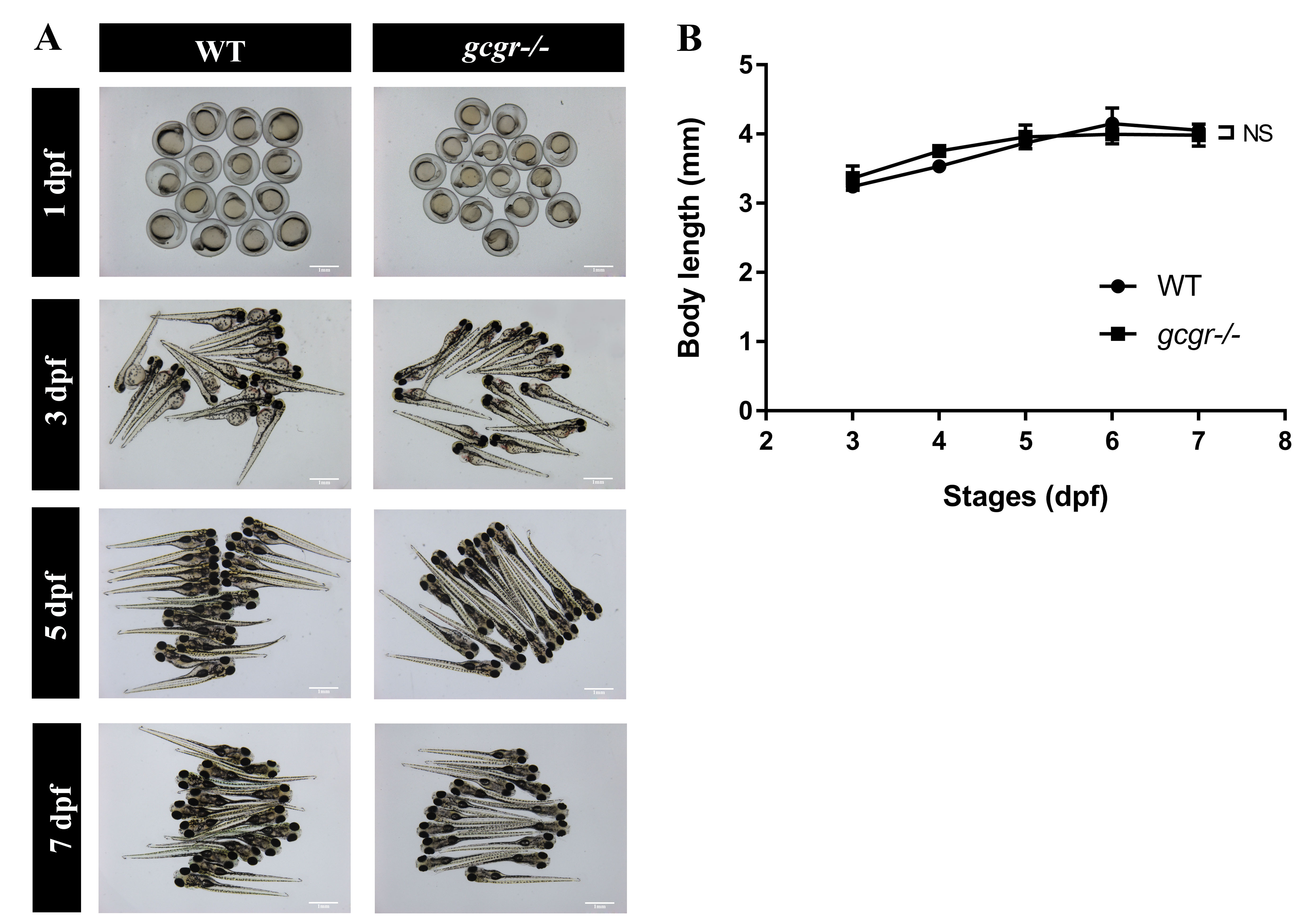

Supplement: Supplementary file 1 [file ijms-21-00724-s001.zip › Supplementary files 20191222/Supplemental Fig1.jpg]
